# Passive Impedance-Matched Neural Recording Systems for Improved Signal Sensitivity

**DOI:** 10.3390/s23146441

**Published:** 2023-07-16

**Authors:** Sk Yeahia Been Sayeed, Ghaleb Al Duhni, Hooman Vatan Navaz, John L. Volakis, Markondeya Raj Pulugurtha

**Affiliations:** 1 Biomedical Engineering, College of Engineering and Computing, Florida International University, Miami, FL 33174-1630, USA; sbeen002@fiu.edu; 2 Electrical and Computer Engineering, College of Engineering and Computing, Florida International University, Miami, FL 33174-1630, USA; galdu001@fiu.edu (G.A.D.); hvata002@fiu.edu (H.V.N.); jvolakis@fiu.edu (J.L.V.)

**Keywords:** neuro-signals, implanted antenna, passive sensor, miniaturized devices, multiband U-slot patch antenna, RF backscattering

## Abstract

Wireless passive neural recording systems integrate sensory electrophysiological interfaces with a backscattering-based telemetry system. Despite the circuit simplicity and miniaturization with this topology, the high electrode–tissue impedance creates a major barrier to achieving high signal sensitivity and low telemetry power. In this paper, buffered impedance is utilized to address this limitation. The resulting passive telemetry-based wireless neural recording is implemented with thin flexible packages. Thus, the paper reports neural recording implants and integrator systems with three improved features: (1) passive high impedance matching with a simple buffer circuit, (2) a bypass capacitor to route the high frequency and improve mixer performance, and (3) system packaging with an integrated, flexible, biocompatible patch to capture the neural signal. The patch consists of a U-slot dual-band patch antenna that receives the transmitted power from the interrogator and backscatters the modulated carrier power at a different frequency. When the incoming power was 5–10 dBm, the neurosensor could communicate with the interrogator at a maximum distance of 5 cm. A biosignal as low as 80 µV peak was detected at the receiver.

## 1. Introduction

Neural recording systems sense and communicate action potentials, local field potentials, post-synaptic potentials, or other relevant bioelectric signals. Sensing such signals can play a key role in the early detection of neurological disorders, identify motor intent, design therapeutic or functional neurostimulation systems, and contribute towards overall advances in neuroscience. The selectivity and sensitivity of the recorded signals are typically enhanced at the expense of the invasiveness of the sensory unit. EEGs (electroencephalograms) are least invasive as the signals are recorded from electrodes on the scalp [1]. These electrodes typically capture the evoked or event-related potentials that are then appropriately decoded with signal processing to extract the features. In one class of applications, these features are translated to understand the motor intent in patients with spinal cord injuries and used to control assistive devices. However, EEGs do not provide a high signal-to-noise ratio and also do not have high-frequency sensitivity to identify the short-term activities with high spatial or temporal resolution.

Direct recording of action potentials with intracortical or depth electrodes provides the highest resolution for brain–computer interfaces (BCI) and therapeutic neurotechnologies [2,3]. These recordings, however, need proximal access to the neurons and are, therefore, the most invasive. They require craniotomy and can often cause neural damage. As a compromise, electrocorticograms (ECoG), which record signals from the surface of the cerebral cortex, provide adequate sensitivity without excessive risk of tissue scarring inside the brain [4]. In spite of this, ECoG recordings are often performed under clinical settings during certain procedures, such as clinical monitoring to identify the epileptic foci [5].

Neural recording systems offer a wide range of electrodes or channels, data rates, bandwidth, power budget, and size or volume depending on the intended applications. High-density microelectrode arrays are often needed to access brain neural circuits with enhanced spatial resolution. Examples of these include intracortical recording to access motor circuits that control the limb movement, mapping to reveal connectivity between neuronal functions or similar large-scale cortical processes. One clinical need where these functions become important is the diagnosis of chronic neurological disorders, such as epilepsy. In this case, the recording units obtain the interictal epileptiform discharges (IED) that occur between seizures. These aid in identifying the pathological area for surgical excision. 

The sensing neural interfaces are fabricated at various scales depending on the channel count and bandwidth. Data rates of 50 Mbps or more are often needed with 32, 64, or 100 channels. In these applications, the power consumption often reaches 100 mW, resulting in a bit energy consumption of 0.1–2 nJ. The recording unit comprises a rigid package that is constructed using PCB (printed circuit boards) substrates with a communication module, an analog signal modulation front-end, and a power module with a battery, and is encapsulated in hermetic packages with electrode feedthroughs. As an example of such units, a system of ~74 cm^3^ and a power consumption of 63.2 mW was designed, out of which 28 mW was consumed by the radio frequency transmitter [6]. To reduce the burden of replacing batteries, rechargeable batteries as well as wireless telemetry were implemented. For example, a recording system for head-stage neural activity with a size of 5.6 × 4.2 × 0.9 cm^3^ on a rigid package with a rechargeable battery was realized [7]. The average power of the system will increase to 100 s of milliwatts when the battery is charging. Even though the system is powered by inductive coils, several problems are foreseen. It has been documented that 400 mW of overall power consumption may negatively affect tissue, as it has been shown that the tissue temperature is increased by 2 °C when more than 40 mW/cm^2^ of dissipated power is applied [8,9].

Because of the extensive power and data telemetry electronics and the associated packaging, wireless neural recording systems are mounted on the top of the skull, whereas the microelectrode arrays (MEA) are typically implanted into the cortex surface. Systems mounted on the skull have a greater degree of design flexibility than the implanted version. They can, thus, accommodate large sizes in terms of the wireless system footprint and significant power consumption. Nevertheless, the intracranial wiring configuration is also accompanied by a wide variety of challenges and issues. It has been observed that the high failure rate is a major concern. Wireless head-stages with implanted microelectrode arrays are not yet safe and robust enough for use in long-term medical treatments [10]. Wirelessly powered neural recording systems have been the primary focus to reduce the need for batteries and associated power conversion [11] and reduce the overall package size. Such power telemetry is often achieved with an inductive link. With advanced coil designs, the inductive link can also be directly integrated onto the IC without compromising the performance [12]. An integrated power rectifier on the IC also minimizes the need for other external components. A CMOS (complementary metal oxide semiconductor) integrated circuit (IC) amplifies, digitizes, and multiplexes the signal, and modulates the backscattered carrier with the serialized data.

Another type of neural recording system consists of distributed individual untethered units that are used to record from only a few sites or a single site. It is possible to miniaturize these down to the size of a few mm, as opposed to larger units of a few cm in size. These are commonly referred to as recording nodes. These provide many unique opportunities for neural recording through multiphysics-based transduction techniques that are derived from ultrasonic, RF, magnetoelectric, or multiferroic excitations [13]. Unlike electromagnetic links that are current-based, these devices often power and communicate through acoustic actuation which scale to the sub-millimeter scale because of their smaller wavelengths compared to microwaves. These techniques have been proven to reduce the power consumption of communication modules as well as reduce the size of the communication modules [14]. Such devices function both in the active mode where the incoming power is rectified to activate a CMOS signal processing circuit, or in a fully-passive mode which merely reflects or backscatters the modulated signal [15].

Multiple coupling modes, such as acoustic, magnetoelectric, or RF backscattering techniques are utilized in fully-passive and implantable neural recording devices. In a typical RF backscattering implementation of this approach, a signal at *F* GHz is transmitted from an interrogator and is received by the implant. The implant upconverts the signal to a *2F* GHz signal, mixes it with a low-frequency low-amplitude neural signal (*f_neuro_*), and sends the modulated signal (*2F* GHz ± *f_neuro_*) back to the interrogator. An antiparallel diode used as a subharmonic mixer, a matching network, and a bypass capacitor are incorporated together on a PCB substrate. Earlier reports demonstrated such passive backscattered sensors with a total size of 15 mm × 16 mm × 1.5 mm [16] With further advances in antenna design, the implant’s size was further miniaturized to 9.5 mm × 8.7 mm × 0.7 mm on a flexible platform [17]. However, in previous studies, implants were typically designed to match the 50 Ω telemetry system. This creates mismatch losses with the high electrode–tissue impedance at the physiological interfaces and limits the sensitivity of the signal. In order to address this limitation, Chen et al. proposed a buffer circuit to convert the high impedance from the electrode–tissue impedance to the matched RF impedance, thereby demonstrating a major advance in signal sensitivity [18]. In this approach, the buffer circuit is integrated with the antenna but with no components to bypass high-frequency signals to improve signal sensitivity.

The applications of wireless passive telemetry systems also extend to wearable medical devices that can sense other biosignals, such as ECG (electrocardiogram), EMG (electromyogram), along with the aforementioned EEG. Passive telemetry can transform today’s obtrusive systems with thick electronic units on the skin to thin and disposable patches. This is because of their zero-power or battery-free operation with minimal signal processing components and simplified topologies. The key challenge with these systems, however, is the need for longer communication distance between the interrogator and the reader. This demands larger interrogation carrier power, which invariably puts a heavier power burden on the interrogator.

Impedance matched circuits and miniaturized antennas that are coupled with low-loss mixers are the key requirements towards enhancing neural recording and broadly all wireless sensor systems. Impedance-buffered electrodes are expected to improve the signal resolution with passive neural recording at low interrogating power. This forms the underlying hypothesis for this work. In order to advance the utility of this approach further, this manuscript describes a miniaturized integrated passive impedance-matching neural recording unit in a thin flexible package with improved sensitivity. A substrate with high permittivity was used to miniaturize the antennas. With these innovations, we capture signals as low as 170 µVpp when the impedance is 33 kΩ. The transmitted biosignals are detectable at a distance of 5 cm between the interrogator and the implanted antenna.

## 2. Neural Recording System Design

**Figure 1:**
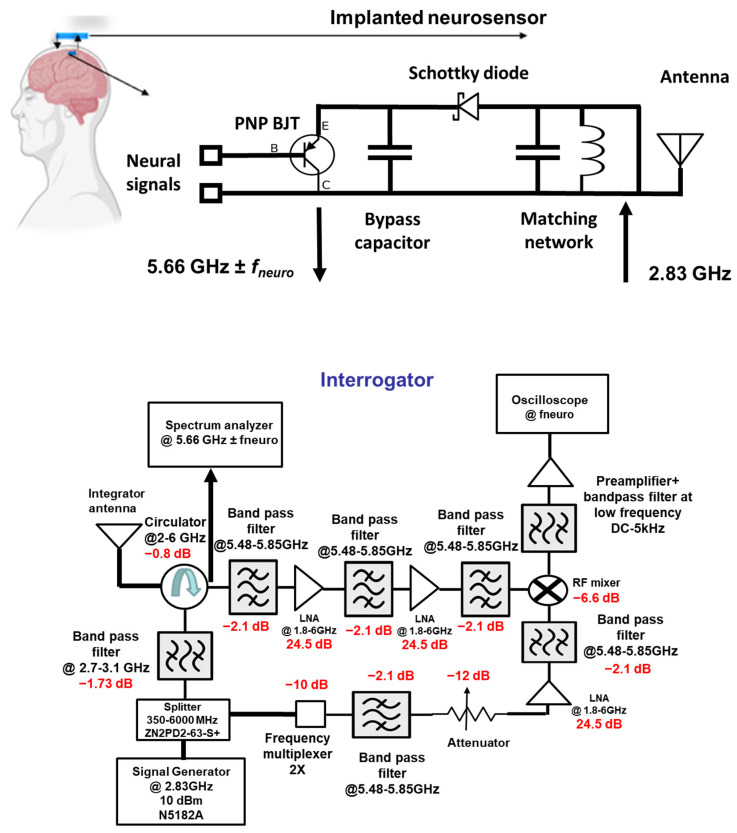
Circuit architecture for the implanted neurosensor block and interrogator.

**Figure 2:**
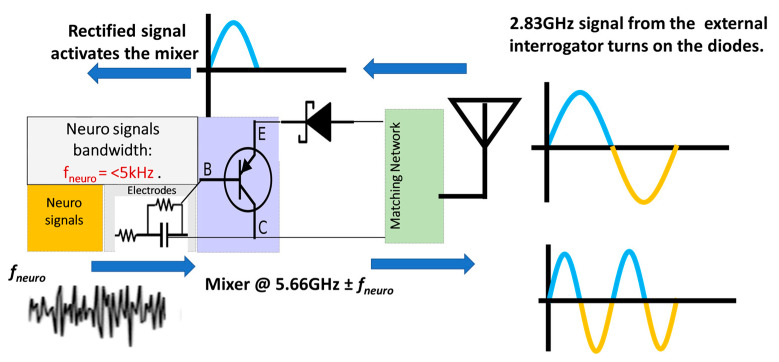
The implanted neural sensor operates in two bands. The diode generates a 2× harmonic and also acts as a mixer. The incoming carrier signal biases the BJT and makes it act like an impedance transformer. The top arrows (pointing to left) indicate the incoming signal. The bottom arrows point to the outgoing signal.

The neural recording system can be divided into two sections: (i) the implant and (ii) the interrogator, as shown in Figure 1. The implanted neurosensor depends on the RF backscattering technique that is widely used in RFID applications. Electrode–tissue impedances are of the order of 10–100 kΩ for practical electrode designs. On the other hand, RF signal chains are designed at 50 Ohms. To deal with high impedance mismatch and DC offset, the backscattering circuit employs a BJT that is biased by an activated diode. The incident signal at *F*1 GHz (typically at ~2.4 GHz) which comes from the interrogator is used to activate the diode in the forward region. The activated or turned-on diode acts as a rectifier circuit to bias the BJT transistor. In the second cycle of the sinewave, the activated transistor and the RF diode act as RF mixers. Thus, it backscatters the *f_neuro_* signals ± 2*F*1 (at ~4.8 GHz) GHz. Figure 2 illustrates the concept of the RF backscattering of the implanted neurosensor. The designs in this paper were implemented at 2.83 and 5.66 GHz.

### 2.1. Passive Sensor

The circuit mechanism is divided into two modes: DC and RF. In DC mode, the interrogator sends a 2.83 GHz carrier signal to activate the implant. Upon receiving the signal, the Schottky diode acts as a rectifier to generate DC and bias the BJT. The BJT collector is connected to the ground while the emitter is connected to the diode in order to upconvert the baseband neuro-signal. The neuro-signal passes through the base when the BJT is biased or activated through the diode. The BJT can be operated either in the forward-active region or in the saturation region. In both scenarios, the signal can move from base to emitter, while the DC offset voltage at the base can be ignored. These unparallel characteristics suggest tolerance to DC offset. As the BJT has a high input impedance and is configured as an emitter follower, it matches the high electrode impedance and converts it into a low impedance. This will ensure that no circuit loading occurs.

**Figure 3:**
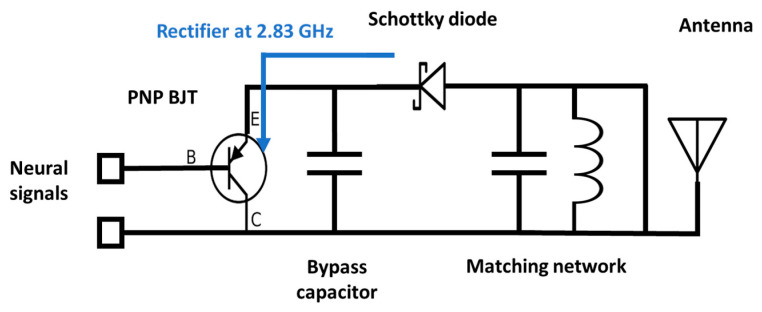

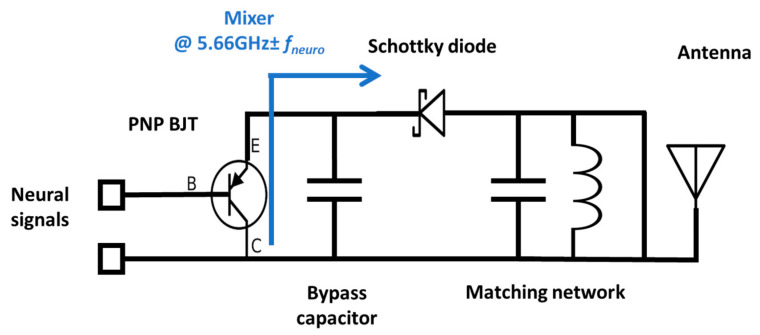
Passive high-impedance matching circuitry. The diode acts as a rectifier (**Top**) and as a mixer (**Bottom**). The BJT behaves as a buffer to reduce the impedance.

In RF mode, the Schottky diode acts as a non-linear mixer, as shown in Figure 3. The 2.83 GHz carrier signal is upconverted with the brain neuro-signal by the diode and generates the second-order component (5.66 GHz ± *f_neuro_*). This second-order product is transmitted back to the interrogator and finally demodulated to retrieve the neural signal in the time–domain.

Thus, the implant features the following key innovations: (a) an implantable antenna for wireless communication, (b) a Schottky diode that operates in DC and RF modes, (c) an inductor for matching diode and antenna impedance, (d) a bypass capacitor to isolate low-frequency signals from high-frequency signals, and (e) a PNP bipolar transistor (BJT) that acts a buffer circuit. The followed components were utilized: a capacitor (Murata Mfg. Co., Ltd., Nagaokakyo, Kyoto, Japan, GRT155R61C105ME01D), diode (Skyworks Solutions, Inc., Irvine, CA, USA, SOT-23 Surface-Mount Mixer and Detector Schottky Diodes—sms7621-079LF), BJT (Onsemi, Scottsdale, Arizona, USA, MMBTH81), and inductor (Murata Mfg. Co., Nagaokakyo, Kyoto, Japan, JELF243C_0021J-01).

In order to achieve the operation in the two frequencies, a unique dual-band antenna that has resonance frequencies at 2.83 GHz and 5.66 GHz was designed. The dielectric substrate of the antenna is based on a ceramic-filled PTFE composite (Rogers RO3010^TM^) with a dielectric constant of 10.2 and a loss tangent of 0.001. We achieved the dual-band operation by cutting a U-shape slot on the patch. Dual-band operation is due to the U-slot’s increase in the current path.

### 2.2. Interrogator

The function of the interrogator is to generate and send a 2.83 GHz carrier signal and demodulate the 5.66 GHz ± *f_neuro_*. It is composed of an RF signal generator, splitter, circulator, multiplying chain, mixer, bandpass filter, low-noise amplifier block, spectrum analyzer, and oscilloscope for signal display and observation. The signal generator produces a 2.83 GHz carrier signal which a splitter routes into the two avenues. The first avenue routes the signal into Port 3 of the circulator and to the interrogator antenna. The second avenue of the splitter routes the signal through the multiplying chain block. With the first avenue, the signal reaches the implant. After signal up-conversion and mixing, the second-order backscattered and modulated signal reaches back to the interrogator. This 5.66 GHz ± *f_neuro_* signal is directed by the circulator to the filter and the amplifying block. This stage has three bandpass filters and two low-noise amplifiers (LNA). As their center frequency is at 5.66 GHz, the undesired second and third harmonic components are filtered out. The signal reaches the mixer with proper signal conditioning by the filter and amplifier stage. At the same time, in the second avenue, the 5.66 GHz signal from the multiplying chain block reaches the LO port of the mixer. Finally, the down-converted low-frequency neural signal is seen on the oscilloscope with proper filtering and amplification at the preamplifier.

## 3. Fabrication

### Fabrication Procedure

**Figure 4:**
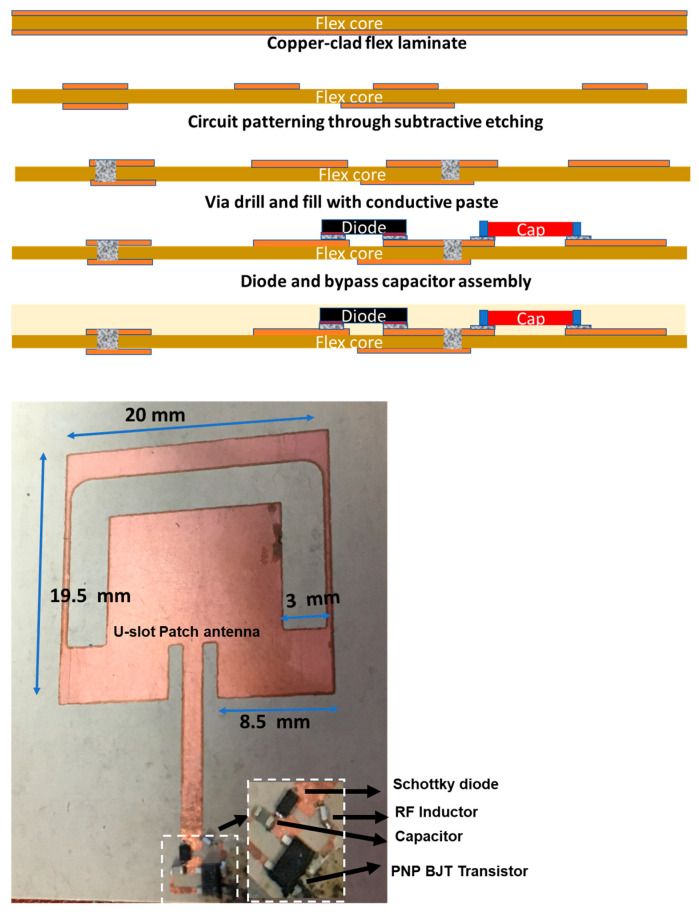
Fabrication procedure and component assembly of the neural sensor (**Top**) and a fabricated sample (**Bottom**). The antenna size is 20 mm × 19.5 mm.

The primary goal is to demonstrate the passive sensor circuit towards implantable applications. Implants need hermetic encasing and feedthroughs with long-term tissue-compatible materials. Hermetic feedthroughs and a biocompatible package are beyond the scope of this paper as only the sensor circuit function is considered in this paper. Rogers RO3010^TM^ laminates were chosen as a substrate, because they have excellent properties at high frequencies, are dimensionally stable, and are effective for circuit miniaturization. Figure 4 illustrates the fabrication process. The sensor traces were obtained by subtractive copper patterning with higher conductivity instead of printed silver traces. To achieve low-cost large-area fabrication, standard dry-film photoresists were used. In order to improve adhesion during copper etching and photoresist patterning, the substrate was cleaned and micro-etched. UV light was used for lithography at a 360 nm narrowband wavelength. Photoresists were developed in a 1% sodium borate solution at 60 °C. Sulfuric acid–peroxide etchant was used to etch copper. The etchant was heated to 60 °C in order to improve the etching rate. The photoresist was removed with acetone to expose the copper traces. On the backside of the substrate, vias were laser drilled and filled with silver paste to interface with the electrodes. Assembly was performed with solders. In order to enhance the electrical and mechanical properties of devices, conductive silver elastomer adhesives were utilized as chip-to-flex interconnection layers. Compared with silver pastes that were loaded with 85 wt.% particles, pastes with 65% of particles were found to exhibit higher mechanical strength and stability during reliability testing in our earlier work and, hence, were used.

**Figure 5:**
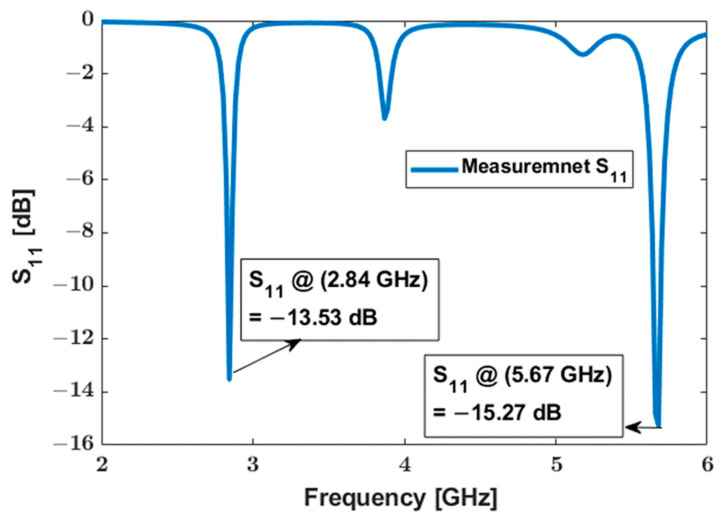
$S_{11}$ measurements from the antenna.

The antenna design is based on a U-slot topology with dual-band operation at 2.83 GHz and 5.66 GHz. The same antenna was also used on the integrator side. An E5071 C Agilent network analyzer was used to measure the $S_{11}$ of the antenna. Measurements showed that the antenna has the desired dual band performance at ~2.8 and ~5.6 GHz, as illustrated in Figure 5.

## 4. Electrical Test, Results, and Discussion

### 4.1. Measurement Setup

**Figure 6:**
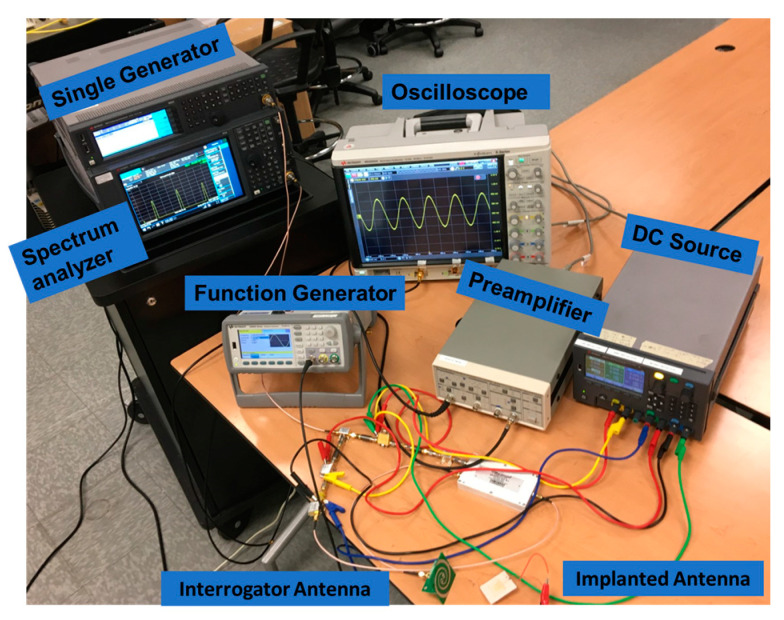
Measurement setup for the passive wireless neural recording system highlighting the receiver circuit and its system components.

The measurement setup for assessing the prototype is demonstrated in Figure 6. A signal generator generates a 2.83 GHz carrier signal in the interrogator. The transmitted power is 5–10 dBm, which is sufficiently below the Schottky diode’s absolute maximum rating (20 dBm). An arbitrary function generator is used with the implant sensor to emulate neural signals with sinusoidal and square waveforms. Since the function generator’s minimum voltage capability is 1 mV, an attenuator is used to generate micro-volt signals. 

### 4.2. Frequency Domain Measurements

**Figure 7:**
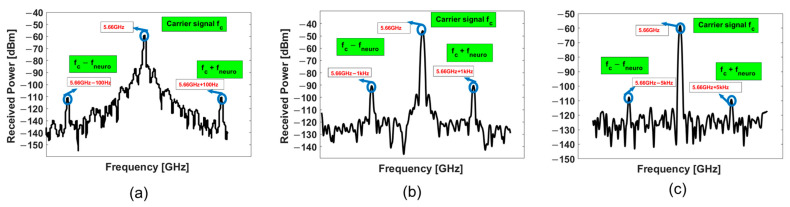
Frequency domain measurements at a 1 cm distance between the implanted antenna and the interrogator. The sideband signal is at (**a**) 100 Hz, (**b**) 1 kHz, and (**c**) 5 kHz *f_neuro_*.

The signal emanates from the function generator (carrier signal at 2.83 GHz) at Port 3 of the circulator. When transmitted through Port 1, this signal is used to activate the passive implanted sensor. The backscattered signal from the implanted antenna at 5.66 GHz flows from Port 1 to Port 2. In the implant, an emulated neuro-signal of 500 µV (−53 dBm) from 100 Hz to 5 kHz is introduced between the BJT base and collector. The received power of the sideband (5.66 GHz ± *f_neuro_*) varies from −116 to −90 dBm. As shown in the interrogator architecture of Figure 1, the frequency domain measurements were taken after the RF circulator (Port 2). Thus, the measurements will be at the backscattered harmonic frequency (5.66 GHz) and also the sidebands at (5.66 GHz ± *f_neuro_*). The measurements were taken at two distances of 1 and 5 cm between the implanted sensor and the interrogator. Figure 7 illustrates the received power at Port 2 of the RF circulator in the interrogator. The power of the carrier signal varies from −46.6 to −60 dBm depending on the neuro-signal.

**Figure 8:**
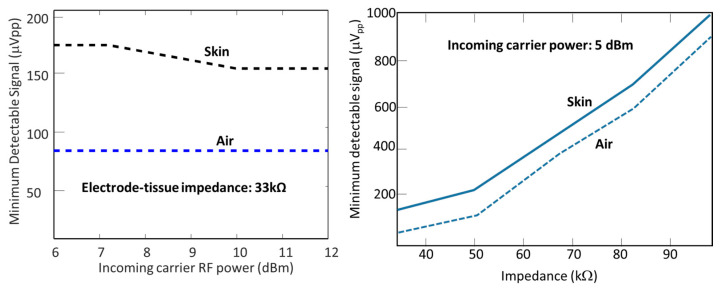
Minimum detectable neural signal as a function of impedance: a neural signal with an amplitude as low as 170 μVpp was detected in the tissue phantom. With the increase in incoming RF power, 150 μVpp was detected from the tissue phantom (**Right**). Weaker 80 μVpp signals were captured in air. In both cases, the electrode–tissue impedance was 33 kΩ (**Left**).

Validation was performed in (1) air and (2) pig (pork) skin of 2 mm thickness, purchased from a meat store. To emulate a high-impedance electrode, resistors from 33–100 kΩ were utilized (see Figure 8). When tested in air, a neural signal with an amplitude as low as 80 μV was detected. However, in a tissue environment, a minimum neural signal amplitude of 170 μVpp was captured. The implant was placed under the 2 mm-thick pig skin. The discrepancy between the signal propagation in air and tissue is due to the additional losses in the tissue medium.

### 4.3. Time–Domain Measurements

**Figure 9:**
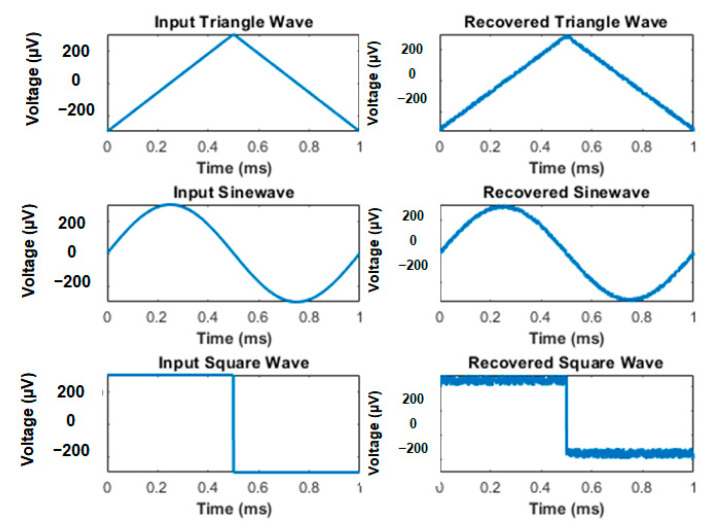
Time–domain signals that are recovered (**right**) from the wireless telemetry with input signals (**left**).

The performance of the neural recording system was validated with time–domain signal retrieval. Time–domain measurements were obtained after the RF mixer, which demodulates the backscattered signal (5.66 GHz) with the 2× multiplied interrogator signal (2 × 2.83 GHz). Different types of emulated neural signals, such as sine, square, and ramp, were generated with an amplitude of 500 µVpp at 1 kHz and retrieved with our interrogator. When the emulated neural signal voltage is low (170 μVpp), digital filtering is required to recover the signal. The recovered noisy signal without filtering is shown in Figure 9.

### 4.4. Discussion and Analysis

The frequency of the signals that can be generated from the brain varies from 1 Hz up to 10 kHz. The voltage of the signals also can be from few µV to mV [11,16]. Table 1 lists the range of the voltage and frequency for specific neuro-signals.

The emulated neuro-signals considered in this work were tested at 100 Hz, 1 kHz, and 5 kHz with voltages ranging from 340–1000 µVrms. To recover the neural signal, it is important to determine the minimum backscattered signal level. Therefore, it is crucial to estimate the noise figure (*NF*) of the interrogator. *NF* is the ratio of the input signal-to-noise ratio and output signal-to-noise ratio of a system and is a measure of the deterioration of a signal caused by a system. The governing Friis equation for noise factor is shown as follows:

(1a) $$F_{\mathrm{cascaded}\;}=F_{1}+\frac{F_{2}-1}{G_{1}}+\frac{F_{3}-1}{G_{1}G_{2}}+\frac{F_{4}-1}{G_{1}G_{2}G_{3}}+\ldots$$

where $F_{1,2,3,..}$ = noise factor for individual components, such as the filter, amplifier, etc., in dB, and $G_{1,2,3,..}$ is the gain for those components. Using Equation (1a), the cascaded noise figure of our system was calculated as 3.8 dB. The noise floor of the system can be calculated as follows:

(1b) $$\begin{array}{c} {\;\mathrm{Noise}\;}_{\mathrm{interrogator}\;}[dBm]=kT+10{log}_{10}\left(B_{IF}\right)+NF_{IN}\\ =-174\left[\frac{dBm}{Hz}\right]+10\log_{10}\left(500Hz\right)+3.8[dB]\\ =-143.2\left[dBm\right] \end{array}$$

where −174 dBm/Hz is the thermal noise power in dBm per 1 Hz of bandwidth at room temperature, and *NF_IN_* is the interrogator noise figure of the system. As shown in Equation (1b), the calculated noise level or floor of the interrogator is −143 dBm. With our experiments, we observed that a 13 dB signal-to-noise ratio (*SNR*) is required to retrieve the neural signal. Therefore, the minimum detectable signal is −130 dBm.

The minimum detectable signal is the sum of the overall system loss and the receiver sensitivity. The overall system loss can be divided into four main components: (1) conversion loss of the implanted mixer, (2) mismatch losses (this mismatch occurs between the mixer and the antenna and also between the BJT transistor and the recording electrode), (3) propagation losses through the tissue, and (4) the propagation loss between the implanted antenna and the interrogator.

Conversion loss is mitigated by working at the optimum power level of the mixer. The detectable signal decreases with increasing RF power because of lower mixer loss. The increase in RF power from 5 dBm to 8 dBm led to the capture of neural signals as low as 150 μVpp from the tissue phantom. However, the transmitter power cannot be increased as it will increase the overall power consumption and introduce other losses. 

Mismatch loss: Another factor that may prevent detecting neural signals weaker than 80 μVpp is the mismatch between the antenna and diode at the carrier frequency. The mismatch losses are addressed at two locations: one is between the antenna and diode, and the other is between the input electrodes and the mixer. The buffer circuit provides this function to reduce losses that come from the impedance mismatch between the electrodes and the mixer. In Figure 8, the minimum detectable voltage is presented as a function of impedance. Signal sensitivity increases with decreasing tissue impedance and lower mismatch. The detectable voltage has a nonlinear dependence on the electrode–tissue impedance. 

Tissue losses: Additional losses arise from the propagation path through the tissue and air. Lossy tissue materials invariably deteriorate the propagation of high-frequency EM waves. Electromagnetic (EM) wave propagation is more complicated in the tissue compared to free space. The lossy property of the tissue leads to the absorption of the wave. An EM wave propagating along the z direction can be defined as follows:

(2) $$E\left(z\right)={Ee}^{-\gamma z}$$

where E stands for the magnitude of the EM wave in the z-direction and γ is the complex propagation constant. Complex propagation constant can be defined as follows:

(3) γ=α+jβ

(4) $$\alpha =\omega \sqrt{\frac{\varepsilon_{0}\mu_{0}\varepsilon_{r}^{\prime }}{2}\left(\sqrt{1+{\left(\frac{\sigma }{\omega \varepsilon_{0}\varepsilon^{\prime }r}\right)}^{2}}-1\right)}$$

(5) $$\beta =\omega \sqrt{\frac{\varepsilon_{0}\mu_{0}\varepsilon_{r}^{\prime }}{2}\left(\sqrt{1+{\left(\frac{\sigma }{\omega \varepsilon_{0}\varepsilon^{\prime }r}\right)}^{2}}+1\right)}$$

where α—attenuation constant, β—propagation constant, $\varepsilon_{r}^{\prime }$—relative permittivity, σ—conductivity, and $\mu_{0}$—permeability for tissues. In short, the complex propagation constant depends on permittivity, permeability, and conductivity. The rise in conductivity makes it a lossy medium where EM waves become attenuated significantly in the lossy medium. Thus, EM waves attenuate with an incremental complex propagation constant in the tissue medium.

Path loss: In air, the distance between the implanted antenna and the interrogator, the gain of the antenna, the incident power, and the medium are the main factors that determine the path loss. Path loss can be determined using the Friis equation for transmission, as shown in Equation (6).

(6) $$P_{r}=P_{t}G_{t}G_{r}{\left(\frac{\lambda }{4\pi D}\right)}^{2}$$

where $P_{r}$ = the received power in watts $P_{t}$ = the transmitted power in watts $G_{t}$ = gain of the transmitted antenna in dBi, $G_{r}$ = gain of the received antenna in dBi, *λ* = the wavelength of the operating frequency in Hz, and *D* is the distance between the implanted antenna and the integrator in m.

The free space path loss can be determined using Equation (7) as follows:

(7) $$Free\;space\;path\;loss[in\;dB]=20\log\frac{4\pi D}{\lambda }$$

**Figure 10:**
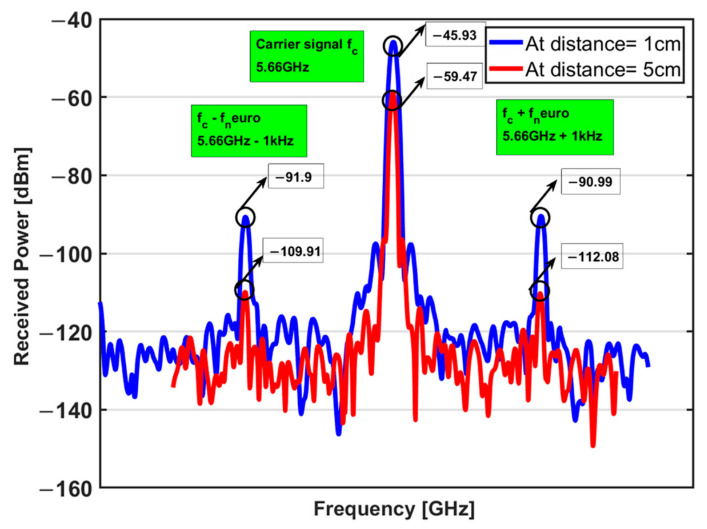
Received power for a 500 µv neuro-signal and 10 dBm carrier signal at a distance of 1 and 5 cm between the sensor and interrogator.

Based on Equation (7), the path loss at 1 and 5 cm will be 8 dB and 22 dB, respectively. Figure 10 illustrates the received power at the carrier frequency (5.66 GHz) and the sideband frequencies [5.66 GHz ± *f_neuro_* (1 kHz)]. The received carrier power decreased by 13 dB when the distance between the interrogator and the sensor was increased from 1 cm to 5 cm. This matches with the expected additional loss of 14 dB from Equation (7). However, the reduction in the received power at the sideband frequencies was 19–21 dB.

## 5. Conclusions

Wireless neural recording systems with high-density circuitry and battery-powered components face constraints because of the need for battery replacement, surgical constraints during implantation that arise from size, and the generation of heat that damages tissues. Passive wireless neural recording devices using RF backscattering eliminate the need for high-density circuitry. However, passive recording units often use non-linear components with relatively low impedance and are, thus, not appropriate for neural recording electrodes as they generate high impedance at the electrode–tissue interface. This causes the system to attenuate the incoming biosignals. In order to address this challenge, the sensitivity of passive neural recording units was improved by implementing a BJT circuit as a buffer, a Schottky diode as a mixer and rectifier, and a bypass capacitor to isolate the signals at high and low frequencies. The sensing circuits were fabricated with high-permittivity Teflon-based dielectric substrates to aid miniaturization, flexibility, long-term stability, and reliability. When the prototypes were tested in a low-loss medium, such as air, neuro-signals with voltages as low as 80 µVpp were detected with an electrode–tissue impedance of 33 kΩ. In the representative phantom tissue, emulated biosignals of 170 µVpp were detected with the same electrode–tissue impedance. As we reach the lower amplitude range of the neural signal, the recovered time–domain signal becomes noisy with high-impedance electrodes.

Mixers with low conversion loss, antenna designs with smaller footprints but high efficiency, and matching networks and receiver circuits with higher signal-to-noise ratio are critical to further improve the signal sensitivity for neural recording applications. These improvements will broaden the applications to other product segments, such as multimodal wearable sensing with disposable on-skin patches that can seamlessly connect to smartphones.

## Figures and Tables

**Table 1:** Typical voltage and frequency ranges of brain signal.

Neural Signals	Voltage [μV]	Frequency [Hz]
Local field potential	10–2000	1–200
Action potential	10–1000	250–10,000

## Data Availability

Data details are available upon email request.

## References

[1] Biasiucci A., Franceschiello B., Murray M.M. (2019). Electroencephalography. Curr. Biol..

[2] Zaer H., Deshmukh A., Orlowski D., Fan W., Prouvot P.-H., Glud A.N., Jensen M.B., Worm E.S., Lukacova S., Mikkelsen T.W. (2021). An intracortical implantable brain-computer interface for telemetric real-time recording and manipulation of neuronal circuits for closed-loop intervention. Front. Hum. Neurosci..

[3] Wang Y., Yang X., Zhang X., Wang Y., Pei W. (2023). Implantable intracortical microelectrodes: Reviewing the present with a focus on the future. Microsyst. Nanoeng..

[4] Ha S., Akinin A., Park J., Kim C., Wang H., Maier C., Mercier P.P., Cauwenberghs G. (2016). Silicon-integrated high-density electrocortical interfaces. Proc. IEEE.

[5] Hill N.J., Gupta D., Brunner P., Gunduz A., Adamo M.A., Ritaccio A., Schalk G. (2012). Recording human electrocorticographic (ECoG) signals for neuroscientific research and real-time functional cortical mapping. JoVE J. Vis. Exp..

[6] Chestek C.A., Gilja V., Nuyujukian P., Kier R.J., Solzbacher F., Ryu S.I., Harrison R.R., Shenoy K.V. (2009). HermesC: Low-power wireless neural recording system for freely moving primates. IEEE Trans. Neural Syst. Rehabil. Eng..

[7] Borton D.A., Yin M., Aceros J., Nurmikko A. (2013). An implantable wireless neural interface for recording cortical circuit dynamics in moving primates. J. Neural Eng..

[8] Wolf P.D., Reichert W. (2008). Thermal considerations for the design of an implanted cortical brain–machine interface (BMI). Indwelling Neural Implants: Strategies for Contending with the In Vivo Environment.

[9] Marblestone A.H., Zamft B.M., Maguire Y.G., Shapiro M.G., Cybulski T.R., Glaser J.I., Amodei D., Stranges P.B., Kalhor R., Dalrymple D.A. (2013). Physical principles for scalable neural recording. Front. Comput. Neurosci..

[10] Barrese J.C., Rao N., Paroo K., Triebwasser C., Vargas-Irwin C., Franquemont L., Donoghue J.P. (2013). Failure mode analysis of silicon-based intracortical microelectrode arrays in non-human primates. J. Neural Eng..

[11] Muller R., Le H.-P., Li W., Ledochowitsch P., Gambini S., Bjorninen T., Koralek A., Carmena J.M., Maharbiz M.M., Alon E. (2014). A minimally invasive 64-channel wireless μECoG implant. IEEE J. Solid-State Circuits.

[12] Kim C., Park J., Ha S., Akinin A., Kubendran R., Mercier P.P., Cauwenberghs G. (2019). A 3 mm× 3 mm fully integrated wireless power receiver and neural interface system-on-chip. IEEE Trans. Biomed. Circuits Syst..

[13] Zaeimbashi M., Lin H., Wang Z., Chen H., Emam S., Gao Y., Sun N. NanoNeuroRFID: A low loss brain implantable device based on magnetoelectric antenna. Proceedings of the 2018 IEEE International Microwave Biomedical Conference (IMBioC).

[14] Rashidi A., Hosseini S., Laursen K., Moradi F. STARDUST: Optogenetics, Electrophysiology and Pharmacology with an Ultrasonically Powered DUST for Parkinson’s Disease. Proceedings of the 2019 26th IEEE International Conference on Electronics, Circuits and Systems (ICECS).

[15] Seo D., Carmena J.M., Rabaey J.M., Alon E., Maharbiz M.M. (2013). Neural dust: An ultrasonic, low power solution for chronic brain-machine interfaces. arXiv.

[16] Lee C.W., Kiourti A., Volakis J.L. (2016). Miniaturized fully passive brain implant for wireless neuropotential acquisition. IEEE Antennas Wirel. Propag. Lett..

[17] Sayeed S.Y.B., Venkatakrishnan S.B., Volakis J.L., Raj P.M. (2023). Miniaturized Fully-Passive Wireless Neural Recording with Heterogeneous Integration in Thin Packages. IEEE Trans. Compon. Packag. Manuf. Technol..

[18] Chen W.-C., Guido K., Kiourti A. (2019). Passive impedance matching for implanted brain–electrode interfaces. IEEE J. Electromagn. RF Microw. Med. Biol..

